# Performance, kinetic, and biodegradation pathway evaluation of anaerobic fixed film fixed bed reactor in removing phthalic acid esters from wastewater

**DOI:** 10.1038/srep41020

**Published:** 2017-02-20

**Authors:** Ehsan Ahmadi, Samira Yousefzadeh, Mohsen Ansari, Hamid Reza Ghaffari, Ali Azari, Mohammad Miri, Alireza Mesdaghinia, Ramin Nabizadeh, Babak Kakavandi, Peyman Ahmadi, Mojtaba Yegane Badi, Mitra Gholami, Kiomars Sharafi, Mostafa Karimaei, Mahboobeh Ghoochani, Masoud Binesh Brahmand, Seyed Mohsen Mohseni, Maryam Sarkhosh, Soheila Rezaei, Hosseinali Asgharnia, Emad Dehghanifard, Behdad Jafari, Alireza Mortezapour, Vahid Kazemi Moghaddam, Mohammad Molla Mahmoudi, Nader Taghipour

**Affiliations:** 1 Department of Environmental Health Engineering, School of Public Health, Tehran University of Medical Sciences, Tehran, Iran; 2 Students’ Scientific Research Center (SSRC), Tehran University of Medical Sciences, Tehran, Iran; 3 Department of Environmental Health Engineering, School of Public Health, Kashan University of Medical Sciences, Kashan, I.R. Iran; 4 Department of Environmental Health Engineering, Aradan School of Public Health and Paramedical, Semnan University of Medical Sciences, Semnan, Iran; 5 Environmental Science and Technology Research Center, Department of Environmental Health, School of Public Health, Shahid Sadoughi University of Medical Sciences, Yazd, Iran; 6 Young Researchers and Elite club, Shahr-e-Qods Branch, Islamic Azad University, Tehran, Iran; 7 Department of Environmental Health Engineering, Faculty of Health, Hormozgan University of Medical Sciences, Bandar Abbas, Iran; 8 Department of Environmental Health, School of Public Health, Sabzevar University of Medical Sciences, Sabzevar, Iran; 9 Center for Water Quality Research (CWQR), Institute for Environmental Research (IER), Tehran University of Medical Sciences, Tehran, Iran; 10 Department of Environmental Health Engineering, Ahvaz Jundishapur University of Medical Sciences, Ahvaz, Iran; 11 Student Research Committee, Ahvaz Jundishapur University of Medical Sciences, Ahvaz, Iran; 12 Department of Accounting, College of Management and Accounting, Yadegar-e-Imam Khomeini (RAH) Shahre Rey Branch, Islamic Azad University, Tehran, Iran; 13 Department of Environmental Health Engineering, School of Public Health, Iran University of Medical Sciences, Tehran, Iran; 14 Research Center for Environmental Determinants of Health (RCEDH), Kermanshah University of Medical Sciences, Kermanshah, Iran; 15 Department of Environmental Health Engineering, School of Public Health, Guilan University of Medical Sciences, Rasht, Iran; 16 Department of Environmental Health Engineering, School of Public Health, Shahid Beheshti University of Medical Sciences, Tehran, Iran; 17 Department of Environmental Health Engineering, School of Public Health, Mashhad University of Medical Sciences, Mashhad, Iran; 18 Social Determinants of Health Research Center, Yasuj University of Medical Sciences, Yasuj, Iran; 19 Department of Environmental Health Engineering, Babol University of Medical Sciences, Babol, Iran; 20 Research Center for Health, Safety and Environment (RCHSE), Alborz University of Medical Sciences, Karaj, Iran; 21 Department of Civil Engineering, Faculty of Engineering, Eastern Mediterranean University, Famagusta, North Cyprus; 22 Department of Occupational Health Engineering, School of Public Health, Tehran University of Medical Sciences, Tehran, Iran; 23 Neyshabur University of Medical Sciences, Neyshabur, Iran; 24 Department of Environmental Health Engineering, Faculty of Public Health, Hamadan University of Medical Sciences, Hamadan, Iran; 25 Department of Environmental Health Engineering, Faculty of Health, Student Research Committee, Tabriz University of Medical Sciences, Tabriz, Iran

## Abstract

Emerging and hazardous environmental pollutants like phthalic acid esters (PAEs) are one of the recent concerns worldwide. PAEs are considered to have diverse endocrine disrupting effects on human health. Industrial wastewater has been reported as an important environment with high concentrations of PAEs. In the present study, four short-chain PAEs including diallyl phthalate (DAP), diethyl phthalate (DEP), dimethyl phthalate (DMP), and phthalic acid (PA) were selected as a substrate for anaerobic fixed film fixed bed reactor (AnFFFBR). The process performances of AnFFFBR, and also its kinetic behavior, were evaluated to find the best eco-friendly phthalate from the biodegradability point of view. According to the results and kinetic coefficients, removing and mineralizing of DMP occurred at a higher rate than other phthalates. In optimum conditions 92.5, 84.41, and 80.39% of DMP, COD, and TOC were removed. DAP was found as the most bio-refractory phthalate. The second-order (Grau) model was selected as the best model for describing phthalates removal.

Environmental pollution by emerging contaminants and hazardous organic compounds is one of the most important concerns in the world due to its adverse effects on the public health and environment^1^,^2^. Phthalic acid esters (PAEs) or phthalates are organic and synthetic compounds which have been listed as emerging, xenobiotic, refractory, and hazardous pollutants^3^,^4^.

PAEs are also considered as potential human health risks, including endocrine disrupting effects^5^, reproductive toxicities^6^, organ damages, birth defects, cancer^3^,^7^, and type 2 diabetes^8^.

Many regulatory agencies including the European Union (EU) and the United States Environmental Agency (USEPA) have classified them as top priority pollutants due to the great concern for PAEs that has arisen in recent years^9^.

In addition to their hazardous health effects, PAEs are ubiquitous in various environments such as natural water bodies, soils, sediments^10^, and industrial and municipal wastewater^11^ due to their widespread global production and distribution^12^, and also due to their ability to leach during manufacturing activities, product use, or even after disposal, because of their physical bounding nature with products^6^,^13^.

Phthalates are used as plasticizer in many industrial products such as packing materials, paints, adhesives^3^, cosmetics^14^, and medical devices^15^ mainly to improve plastic polymers’ flexibility and toughness.

Meanwhile, industrial wastewater discharge led to leaching of high concentrations of PAEs (as high as 500 mg/L) to the environment; therefore, outlet wastewater has to be remediated before discharging^1^,^3^.

Although it has been found that PAEs degradation by physico-chemical processes including photodecomposition and hydrolysis occurs slowly, bioremediation can play an important role in their bio-degradation with harmless by-products^9^,^16^.

On the other hand, long alkyl (ester) chains phthalic acid esters with high molecular weights, such as dioctyl phthalate (DOP) and di(2-ethylhexyl) phthalate, (DEHP) are refractory to biodegradation. Therefore, based on environmental considerations, short alkyl chains PAEs including diethyl phthalate (DEP), dimethyl phthalate (DMP), and phthalic acid (PA) can be good substitutes for them^17^.

Economical wastewater disposal is an important issue^18^, and among different biological technologies for industrial wastewaters treatment with high chemical oxygen demand (COD) concentrations, anaerobic processes can be the most economical^19^.

Furthermore, in recent years, the number of anaerobic industrial wastewater treatment plants has shown an upward trend, and they have advantages including less need for nutrients and energy, and low sludge production^19^.

Among different anaerobic wastewater treatment processes, biofilm-based reactors, especially fixed bed reactors such as anaerobic fixed film fixed bed reactors (AnFFFBR), are one of the early developments in wastewater treatment and have been used successfully^19^,^20^.

In addition to benefits of anaerobic bioreactors mentioned above, the AnFFFBR and other earlier anaerobic attached growth processes have additional advantages over suspended growth, which are related to using immobilized cells on the carrier (biofilm). These advantages include higher resistance to environmental shock (toxic compounds, pH, and temperature), greater population variation, and a higher substrate utilization rate^19^,^21^.

As mentioned before, due to their adverse effect and extensive application, the biodegradation of PAEs should be evaluated and compared for finding the best eco-friendly phthalate whose wastewater can be biodegraded better than others. As highlighted in the previous paragraph, short chains PAEs can be biodegraded with higher rates compared with phthalates with long chains. Therefore, the major objective of this study was to investigate and compare diethyl phthalate (DEP), dimethyl phthalate (DMP), diallyl phthalate (DAP), and phthalic acid (PA) biodegradations to find the best suitable phthalate which can be suggested for industrial applications. It should be noted that there is very little or even no information on DAP biodegradation, especially in anaerobic conditions. These four selected phthalates have molecular weights lower than 250 g/mol and can be considered as short-chains phthalates. Moreover, their solubility is significantly higher than other phthalates^17^. However, over the past decades, many environmental engineers tried to find a feasible and simple way for understanding bioreactors’ behavior with different substrates. Although, a number of studies have been presented for the mentioned biotechnological issues, most of them only focused on removal efficiencies, and others merely presented the kinetic coefficients^22^,^23^,^24^. Furthermore, there is still a research gap for evaluating and interpreting all the mathematical modeling, kinetic coefficients, and biomass concentrations (or other bioreactor responses) at the same time. Thus this study has been conducted in order to investigate the relations among these three parameters and present an applicable way for interpreting, evaluating, and controlling the bioreactor performance.

Metabolic intermediates which had been formed during enzymatic bio-reactions and biogas production were analyzed for all selected phthalates. Finally, interactions among various parameters and bioreactor performance, such as biofilm mass, which have not been well studied before, were examined in detail.

## Methods

### Set-up and operation of bioreactor

A laboratory scale rectangular cube Plexiglas reactor, with length and width of 10 cm, height of 70 cm, and operating volume of 6 L, was used. The top of the bioreactor was equipped with gas collector and connected to a gas meter. The heater was placed in a bioreactor chamber to keep the temperature around 25 °C. The bioreactor was filled with high-density polyethylene carriers with 535 m^2^.m^−3^ and 0.95–0.98 g.cm^−3^ of active surface area and density, respectively, as a fixed bed for biofilm growth of microbial mass. Total available surface area in AnFFFBR was 1.6 m^−2^.

Synthetic wastewater was fed continuously to the bottom of the AnFFFBR from the storage tank through a dosing pump (Etatron-Italy).

In order to have a COD/nitrogen/phosphorous ratio of 350/5/1, NH_4_HCO_3_ and NH_4_Cl in the same portion were used for the nitrogen source, and KH_2_PO_4_ was used for the phosphorous source as nutrients.

Furthermore, synthetic wastewater had other constituents: 90 and 14 mg/L of MgSO_4_.7H_2_O and CaCL_2_.2H_2_O, respectively, and 0.3 mL/L of trace solution. For preparing the trace solution each of the following compounds were dissolved in one liter of distilled water: 0.03 g of CuSO_4_.5H_2_O, 0.15 g of CoCl_2_.6H_2_O, 0.12 g of ZnSO_4_.7H_2_O, 0.18 g of KI, 0.12 g of MnCl_2_.H_2_O, 0.15 g of H_3_BO_3_, 0.06 g of Na_2_MoO_4_.2H_2_O, 10 g of EDTA, and 1.5 g of FeCl_3_.6H_2_O^25^. NaHCO_3_ was employed to adjust the pH of 7.5 ± 0.2 in the bioreactor.

The anaerobic sludge of municipal wastewater treatment plant (MWTP) was utilized as a seed to set up the AnFFFBR. For adaptation, initially 600 mg/L of glucose was used as the sole carbon source. After 67 days, when the steady-state condition was attained (the soluble COD removal variations were below 2% at least for 5 days, and one-way ANOVA test was used to confirm that these variations were not statistically significant), the second stage was started and the phthalic acid (PA) was gradually added. At this stage, 150 mg/L of glucose was replaced with 75 mg/L of phthalic acid (PA) (influent wastewater had 75 mg/L of PA and 450 mg/L of glucose). The substrate substitution was continued with a similar rate for three further stages in sequence, until only the PA remained as the substrate (300 mg/L of PA and 0 mg/L of glucose). A similar method was applied for other selected phthalates with the order of DMP, DAP, and DEP in sequence. It should be noted that the initial acclimation sludge had been kept in parallel and in similar conditions (600 mg/L of glucose and a similar composition of nutrient and trace elements) for further AnFFFBR seeding, when the new phthalate substituted the previous one (e.g. when the PA was replaced with DMP). A similar method applied for other phthalates. In the start-up period, an additional dosing pump was used to recycle washed-out sludge from the settling tank.

### Analytical methods

The extent of mineralization of phthalates was monitored by total organic carbon (TOC) analyses. After filtering samples with a 0.45 μm filter, they were analyzed by TOC-Vcsh analyzer (Shimadzu, Japan).

Soluble chemical oxygen demand (in present study indicated as COD) and characteristics of biomass, including volatile solids (VS) and total solids (TS) were analyzed periodically according to the analytical methods as presented in standard methods^26^. The biofilm was removed within two steps: initially, it was removed from carriers physically; then, the ultrasonic treatment, which is described in Chu and Wang’s (2011) study^27^, was used for the complete removal of the remaining biofilm.

In order to quantify the dry weight of the attached biofilm, 15 pieces of carriers were taken from the AnFFFBR (new carriers were replenished), and then the detached biofilm was dried at 105 °C and subsequently weighted.

For phthalates’ analysis, 10 mL of the effluent sample was filtered through a glass fiber filter with a 0.7 μm pore size, and then extracted with 2 mL of n-hexan. Finally, the extracted sample was analyzed by gas chromatograph (GC) and equipped with a capillary HP-5 column and flame ionization detector (FID).

Temperature program and other conditions of GC were as follows: the initial oven temperature of GC was set at 70 °C and kept for one minute, and then was raised at a rate of 10 °C/min until the final temperature of oven reached 250 °C, and then it was held for 2 minutes. The temperatures of the detector and injector were set at 260 and 250 °C, respectively. Nitrogen was employed as a makeup and carrier gas.

Naphthalene was selected as the internal standard, and phthalates concentration in samples were determined by comparing them to their calibration curves which were prepared at five points. The injection volume of sample was 2 μL.

Analytical measurement of methane and other gasses in biogas were performed according to the study by Lay *et al*.^28^.

Detecting intermediates after metabolic reactions were performed through gas chromatograph (GC) and liquid chromatography (LC) which were equipped with single and two mass spectrometer (MS) detector(s), respectively (GC-MS and LC-MS/MS).

After 1 g of freeze-dried sample was ground and extracted by 10 mL of n-hexan solution, the same method of measurement of phthalates in effluent was used for quantifying phthalates’ concentration in sludge.

### Mathematical kinetics modeling

Mathematical models and critical kinetic parameters (e.g. sludge yield coefficient and overall reaction rate) are important variables for predicting bioreactors performance and designing biological wastewater plants.

First order, second order, and Stover-Kincannon, which are three common mathematical substrate utilization models, are applied in the present study to predict the AnFFFBR performance and evaluate the biodegradability of selected phthalates. When the steady-state condition is attained, if the first order model prevails, substrate removal rate can be predicted by Eq. (1):


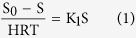


where K_1_ is the first order constant and expressed as (d^−1^); S and S_0_ are substrate concentrations in effluent and influent, respectively; Q is the hydraulic loading rate (L.d^−1^); and HRT is hydraulic retention time (day).

For predicting substrate removal rate in biofilm based reactors, Stover-Kincannon model was modified and linearized as Eq. (2).


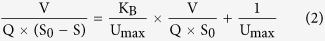


where U_max_ is the maximum substrate removal rate (mg COD/L.d), K_B_ is the saturation value constant (mg/L.d), and V is the reactor volume (L).

Under steady-state conditions, if the second order model prevails, substrate utilization rate can be predicted by Eq. (3) and, subsequently, second order coefficient (K_G_) which is expressed as (d^−1^) can be determined by Eq. (4).


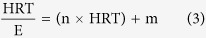



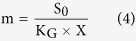


where (m, d^−1^) and (n, dimensionless) are second order model constants and can be calculated from the intercept and slope of plotted line of 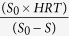 versus HRT. (X) is the biomass concentration and expressed as (mg volatile suspended solids (VSS.L^−1^)). (E) is removal efficiency of utilized substrate in bioreactor (dimensionless).

Half saturation constant (K_s_) which is expressed in (mg.L^−1^) and overall reaction rate (K) which is expressed in (d^−1^) for attached growth bioreactors under steady-state conditions (ds.dt^−1^ = 0) can be determined by combining Eqs. (5) and (6) which are mass balance and Monod equations, respectively:


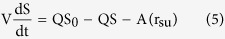



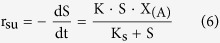


where V is the reactor volume (L); X_(A)_ represents attached biomass per area of fixed bed (g VS.m^−2^); A is the available surface area (m^2^); and r_su_ is the substrate utilization rate (mg.m^−2^.d^−1^). When steady-state conditions are attained, the rate of substrate concentration change in Eq. (5) is negligible (ds.dt^−1^ = 0), and Eqs (5) and (6) can be combined and rearranged as Eq. (7). Substituting 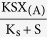 from Eq. (6) with r_su_ from Eq. (5) will yield Eq. (7) as follows:


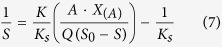


(K) and (K_S_) kinetic constants can be calculated from slope and intercept of linear regression of Eq. (7), respectively.

Biomass yield coefficient (Y) and biomass decay rate (K_d_) which are expressed in (g VS produced/g substrate utilized or dimensionless) and (d^−1^) can be determined by using biofilm mass balance equation and Monod growth kinetic, which can be written as Eqs (8) and (9).


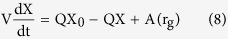



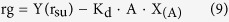


When steady-state conditions (dX.dt^−1^ = 0) are attained, Eq. (10) which is a linearized equation can be obtained by substituting 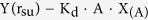 from Eq. (9) with r_g_ from Eq. (8), as follows:


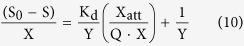


Subsequently, (Y) and (K_d_) can be determined by linear regression of plotted line of (S_0_-S/X) versus 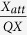. X_att_ which represents attached biofilm mass is expressed as (g VSS) and calculated by multiplying (A) and (X_A_).

Finally, maximum specific growth rate coefficient (μ_m_) can be determined by multiplying (Y) and (K) coefficients, Eq. (11)^29^:


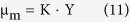


## Results and Discussion

### AnFFFBR performance evaluation

Anaerobic fixed film fixed bed reactor performance was evaluated under different substrates including DAP, DEP, DMP, and PA, and different hydraulic retention times (HRTs). In order to evaluate removal efficiencies and kinetic behavior of mentioned substrates, effluent concentrations of phthalates, COD, and TOC were analyzed as bioreactor responses. Moreover, attached and sloughed biofilm masses were measured. The experimental results for AnFFFBR performance are shown in Tables 1 and 2.

The experimental results for all four study steps (A to D) indicate that phthalates’ removal increased from study runs 1 to 5, as HRT increased. Moreover, COD and TOC removal of all the substrates showed similar behavior. It could be due to higher bio-availability of biofilm mass to the substrates, and providing a higher contact time for secreted enzymes from microbial mass^3^. According to the comparison of AnFFFBRs’ performance in term of phthalates, COD, and TOC removal, it can be concluded that this bioreactor has a better performance for phthalates’ removal than for their mineralization. For instance, in study phase (B-1) with DMP as substrate, the DMP removal was 71.26% which is considerably higher than 46.5% of TOC removal. In addition, with increasing HRT, the difference between phthalates and their mineralization decreased (e.g. with DMP as the substrate, the difference between DMP and its TOC removal decreased from 24.76% (study phase B-1) to 12.11% (study phase B-5)). This demonstrates that the performance of AnFFFBR is significantly dependent on HRT. Higher phthalates’ removal rather than their mineralization may be related to their benzene (aromatic) ring which is more bio-refractory than their side alkyl (ester) chains. It is in good agreement with the experimental observations of Spagni *et al*. which reported that the formed aromatic compounds are refractory to anaerobic digestion^30^. In addition, this can be supported by other studies which stated that aromatic compounds are refractory^31^,^32^,^33^.

Among the four selected phthalates, the maximum removal efficiency was observed with DMP as substrate, which was 92.5, 84.41, and 80.39% for DMP, COD, and TOC removal (study phase B-5), respectively. On the other hand, the minimum removal efficiency was observed with DAP as substrate, which was 59.43, 51.86, and 39.74% for DAP, COD, and TOC removal (study phase A-1), respectively. One-way ANOVA analyses for COD, TOC, and phthalate removal efficiencies originated from different phthalates showed that removal efficiencies of the mentioned parameters for DEP, PA, and DMP were statistically different from those of DAP in all study phases (P-value < 0.05). It should be highlighted that, although one-way ANOVA indicates that some study phases for DMP and PA removal were statistically different (e.g. the COD and phthalate removal in HRTs of 24 and 30 h for DMP were statistically higher compared with PA), there were other study phases in which the performances of bioreactor were close. However, further comparison of these two phthalates should be assessed with their kinetic coefficients. According to the obtained results, the AnFFFBR with DMP and PA had better phthalates removal performance than with DEP and DAP. By comparing physico-chemical properties of selected phthalates as presented in previous studies^17^,^34^ a correlation was observed between alkyl chains’ length, octanol-water partition coefficient (K_OW_), and molecular size of these phthalates and their removal. It can be concluded that phthalates with shorter chain lengths, lower molecular sizes, and lower octanol-water partition coefficients can be removed in a higher rate. Although the PA has the lowest molecular weight and K_OW_ and the shortest alkyl chains, the DMP removal was performed in a slightly higher rate (except for study phase B-1 compare to D-1) which may correlate with the higher solubility of DMP (4200 mg/L) compared with PA (625 mg/L)^17^,^34^. DMP solubility (which is the highest solubility among selected phthalates) can lead to higher diffusion of this substrate and consequently higher bio-availability for biofilm mass to utilize it. Despite the fact that the solubility of these phthalates can influence bioreactor performance, comparing biodegradation of DEP which has a higher water solubility (1000 mg/L) and PA (which has a higher removal rate) can demonstrate that alkyl chains’ length has an independent effect on phthalates’ removal. It should be noted that for most of the study phases, one-way ANOVA results for phthalate and TOC removal efficiencies originated from DMP and PA were statistically different when compared with DEP (P-value < 0.05).

The solids’ retention time (SRT) which represents the average presence time of the microbial mass in the bioreactor^19^, increased as HRT increased (e.g. from 18.7 to 30.53 (day) with PA as substrate). This increase can be another reason for improving phthalates’ removal in higher HRTs. Moreover, the obtained SRTs for this study were considerably more than the SRTs needed for wastewater treatment in other conventional treatment plants such as activated sludge, due to the type of biofilm growth which is attached in this study^19^. It has been reported that typical SRT values in the range of 4 to 10 days can result in carbon oxidation in full scale aerobic wastewater treatment plants, while its values should be long enough for removing refractory compounds^35^. This can demonstrates the AnFFFBR has significant ability to tolerate and remove all the selected phthalates.

In addition, changes in attached biofilm mass (VS, mg) in all phases of the study were lower than the organic loading rates (e.g. with DMP as substrate, the attached mass (mg VS) only decreased nearly 1.78 times, while the organic loading rate (OLR) reduced three times from study phase B-1 to B-5).

The rate of adsorbed phthalates’ concentration to the sloughed biofilm (produced sludge) is also another important factor which must be considered, especially whenever the sludge is applied for soil conditioning. As PAEs have high K_OW_, they tend to accumulate in soils or other organic constitutes. According to the experimental results, all phthalates and especially DAP enriched in sloughed biofilm. The maximum concentration of phthalates in sloughed biofilm belonged to DAP as 11.9 (mg DAP/g TSS) in study phase A-1. Higher K_OW_ of DAP can result in higher hydrophobicity of this phthalates which consequently leads to more biosorption of this phthalate to microbial mass^17^. The maximum adsorbed phthalates was observed for DAP as 11.9 mg DAP/mg sloughed biofilm (study phase A-1) and, by contrast, the minimum concentration was observed for PA as 1.8 mg PA/mg sloughed biofilm. It should be noted that all adsorbed phthalates’ concentrations were significantly higher than the amounts reported in previous studies (e.g. the highest PAEs concentration in primary and secondary sludge was reported as 1250 mg PAEs/Kg sludge^36^). It indicates that all produced sludge of AnFFFBR must be digested before disposal or application to the land.

The main gasses detected in collected biogas for all selected phthalates were similar and included methane, carbon dioxide, hydrogen, and hydrogen sulfide.

The biogas analysis results represent that methane production rate and its concentration (or its percentage) in biogas were very sensitive to HRT and OLR, and in lower HRTs and higher OLRs methane production rates were considerably reduced for selected phthalates. Reducing methane yields in higher OLRs (lower HRTs) might be related to the toxic effects of phthalates on methanogen bacteria, because similar results were recorded for all of the selected phthalates. Moreover, according to the obtained results, in lower HRTs and consequently higher hydraulic loading rates, the SRTs decreased significantly, which can produce unfavorable conditions for growth and activity of methanogen bacteria. It is well known that methanogens are more sensitive to environmental conditions and have slower growth rates^19^,^37^,^38^,^39^. In addition, it has been reported that increasing OLR of some specific wastewaters can decrease the specific activity of methanogen bacteria^40^,^41^. Similar results have been reported by Chaisri *et al*. for anaerobic filter, and higher organic loading rates decreased methane production rates^42^.

The maximum methane production rate for DAP, PA, DEP, and DMP were observed as 0.35, 0.4, 0.43, 0.46 L CH_4_/g COD_rem_ with HRT of 36 h, respectively. The obtained methane yields for study phases (B-5), (C-5), and (D-5) were above the theoretical methane production which is usually expected to be around 0.35 L CH_4_/g COD^43^. Considerable obtained methane yields can be as the result of high SRTs observed for this bioreactor, which can improve the presence of slow-growing bacteria such as methanogens^44^. It has been known that biofilm-based processes are useful, especially when slow-growing bacteria have to be kept in the bioreactor^45^. Moreover, the phenomenon of bacterial cell death and subsequently lysis which can be intensified in higher SRTs, especially in inner layer of biofilm, may result in releasing more substrates as C_5_H_7_NO_2_^19^ which can be used by other active bacteria and consequently produce more biogas. These hypotheses can be confirmed, since study phases (B-5), (C-5), and (D-5) which were operated in maximum HRTs, had the lowest volatile suspended solids (VSS_e_) in their effluents as 36, 48, and 46 mg.d^−1^, respectively. There are other studies which had reported that co-digestion of wastewater with volatile solids (VS) can significantly increase methane production rates. These low VSS_e_ can demonstrate that more bacterial remained tissues were converted to final and mineralized products such as methane, carbon dioxide, and other inert minerals. As an example, Tezel *et al*. reported that co-digestion of four different samples of wastewater with initial COD concentrations of 0.6 to 1.53 g COD/L and VS of 0.1 to 0.71 g VS/L can lead to methane production rates up to 0.98 (L CH_4_/g VS added)^46^. Furthermore, there are other studies that reported methane yields between 0.41 and 0.66 L CH_4_/g COD for anaerobic wastewater treatment^47^,^48^,^49^.

Additionally, in most study phases, methane production rates with DAP as substrate were considerably lower than in comparison with other substrates. As a result, according to the potential of biogas production, DMP can be considered as a suitable substrate due to its higher methane production rate.

**Figure 1:**
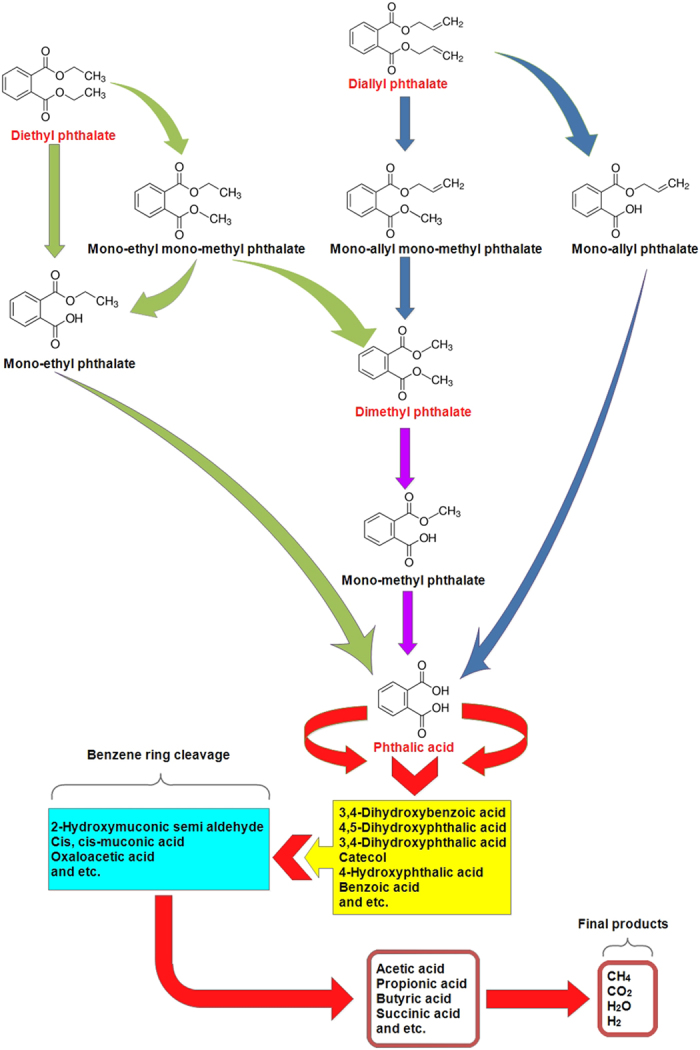
The proposed bio-degradation pathways of selected phthalates.

The proposed biological pathway of phthalates is presented in Fig. 1. A total of four primary intermediates were identified during DAP biodegradation including mono-allyl phthalate, dimethyl phthalate, mono-methyl phthalate and PA. According to the results, DAP was initially metabolized to mono-allyl phthalate and then the bioreaction moves forward to produce phthalic acid. This could be caused by enzymatic ester hydrolysis which commonly known as a de-esterification (Ahuactzin-Pérez *et al*., 2016; Liang *et al*.^50^, Detecting trace amount of DMP and mono-methyl phthalate can be as a result of second biodegradation route which is termed as de-methylation (it should be noted that mono-allyl mono-methyl phthalate was not detected)^50^. Similar observation was reported by Amir *et al*. where DMP and dibutyl phthalate (DBP) were observed as by-products of diethyl hexyl phthalate (DEHP) biodegradation during sludge composting^51^. However, many studies reported that the de-esterification is the main route in phthalates biodegradation^50^.

Similar pathway observed for DEP biodegradation and mono-ethyl phthalate, mono-ethyl mono-methyl phthalate, DMP, mono-methyl phthalate, and PA were detected as its primary metabolites. In addition, mono-ethyl phthalate and PA were the main by-products and this can indicate that de-esterification is the main route of DEP biodegradation.

Furthermore, DMP metabolite analyzing indicates that removing its side chains (de-esterification) is the only route of biodegradation as the mono-methyl phthalate and phthalic acid were detected as its only primary by-products.

Analyzing by-products of DAP, DEP and DMP biodegradation can demonstrate that these compounds have similar pathways as detachment of side alkyl chains (de-esterification) is the main route for their biodegradation and phthalic acid was detected as a central metabolite in their biodegradation.

In addition, phthalic acid biodegradation led to producing 3,4-dihydroxybenzoic acid (protocatechuic acid), catechol, benzoic acid, 4-hydroxyphthalic acid (4-hydroxyphthalate), 4,5-dihydroxyphthalic acid (4,5-dihydroxyphthalte), 3,4-dihydroxyphthalic acid (3,4-dihydroxyphthalate), etc. Some of these detected metabolites including benzoic acid, 3,4-dihydroxyphthalate, catechol and 4,5-dihydroxyphthalate were similarly reported by other studies conducted for biological removal of phthalates^50^,^52^. The biodegradation continued with benzene ring cleavage of mentioned metabolites produced from PA decomposition, and formed other by-products such as 2-hydroxymuconic semi aldehyde. Then, the pathway continued by producing common volatile fatty acids (VFAs) of anaerobic wastewater treatment including acetic acid, propionic acid, butyric acid, etc. These detected VFAs are common by-products of anaerobic biodegradation of organic compounds^19^. Eventually, acetogens consume these VFAs and produce acetate, hydrogen, carbon dioxide, etc, which are the precursors of methane production^19^,^53^. Finally, biodegradation pathway ended with producing carbon dioxide, methane and hydrogen which were detected in biogas as previously stated. These final products indicate that biological removal of phthalates with AnFFFBR can be considered as a reliable, safe, and eco-friendly treatment. Furthermore, it should be mentioned that other non-phthalate metabolites such as VFAs and methane can be produced at each step of biodegradation pathway and not solely at it end.

### Kinetic evaluation of phthalates removal

**Figure 2:**
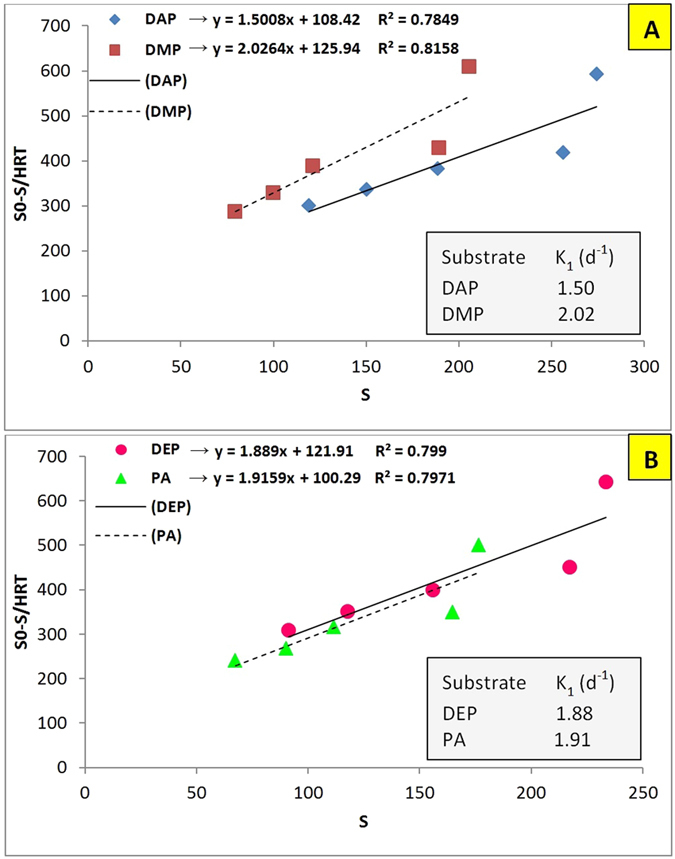
First order model for removing (**A**) DAP and DMP; and (**B**) DEP and PA in AnFFFBR.

The first order model for selected phthalates has been shown in Fig. 2. According to the obtained first order coefficients (K_1_), the COD removal for selected phthalates increased stepwise in the following order: DAP, DEP, PA, and DMP, which confirmed the obtained results; nevertheless, this model still cannot be applied due to its relatively low degree of precision (nearly R^2^ < 0.8).

**Figure 3:**
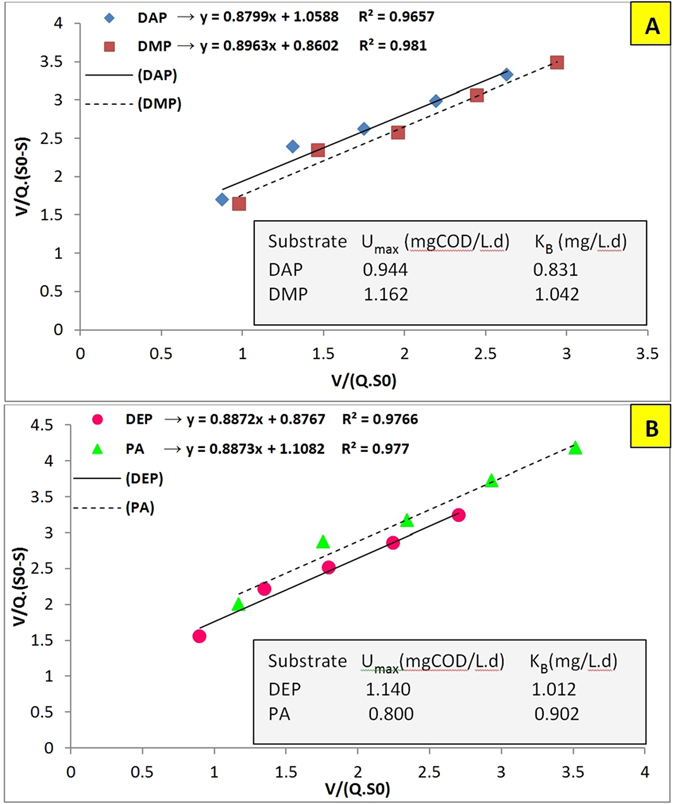
Stover-Kincannon model for removing (**A**) DAP and DMP; and (**B**) DEP and PA in AnFFFBR.

In order to calculate the maximum substrate removal rate (U_max_) and saturation value constant (K_B_), Eq. (2) was plotted in Fig. 3. The maximum U_max_ was observed for DMP as 1.162 mg COD/L.d which demonstrates that DMP has the highest biodegradability. Moreover, this model has high coefficients of determination for all selected phthalates (0.96 ≤ R^2^ ≤ 0.98), which means its use is preferred to the first order model for predicting phthalates’ removal in AnFFFBR; but the U_max_ value for PA (0.8 mg COD/L.d) was lower than that of other phthalates, which contradicts the obtained results. This reflects the disadvantage of Stover-Kincannon model when this model is applied for biofilm reactors. It should be noted that this model does not involve the effect of microbial mass and had been developed solely based on organic loading rate^3^. In this regard, it will be hard to design biofilm base reactors without considering the impact of available surface area and microbial mass, and it should only be used for predicting bioreactors’ substrate removal rate.

**Figure 4:**
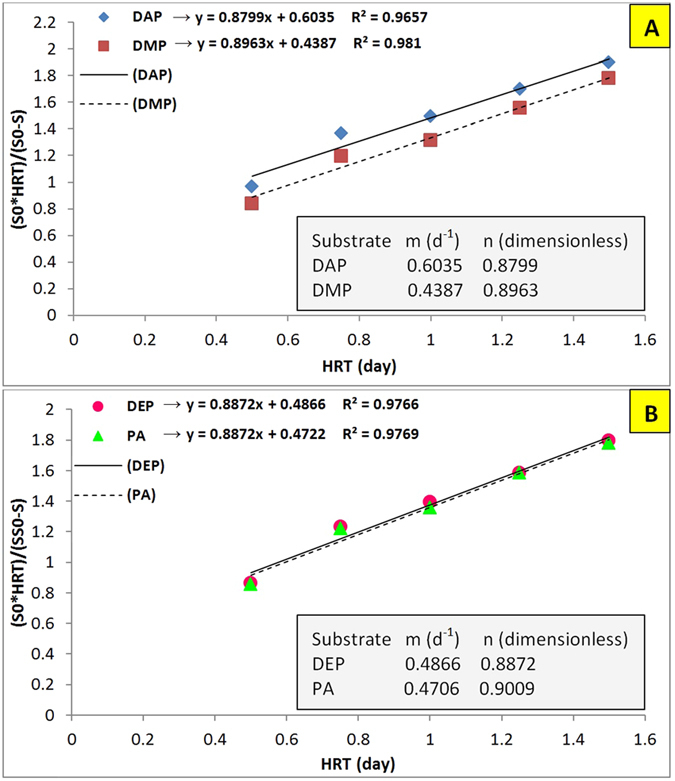
Grau (second order) model for removing (**A**) DAP and DMP; and (**B**) DEP and PA in AnFFFBR.

Figure 4 represents the graph plotted for calculating second order coefficient (K_G_). Considering the high coefficients of determination obtained for all phthalates’ removal (R^2^ > 0.96), this model can be used for predicting and describing AnFFFBR performance with a high degree of precision. Moreover, the obtained K_G_ which represents the substrate utilized per each unit of microbial mass was in good accordance with the obtained results. Based on the K_G_ values which are shown in Table 3, DMP had the highest biodegradability and utilization in AnFFFBR. The biodegradability of phthalates were measured in the following order: DMP, PA, DEP, and DAP, and K_G_ values were 3.52, 3.11, 3.00, and 2.86 (d^−1^), respectively, in optimum conditions (study run 5 for all phthalates).

The obtained K_G_ values of all substrates were raised significantly as HRT increased. This may be the result of higher bioavailability of microorganisms to the substrate, which consequently increases the substrate biodegradation.

**Figure 5:**
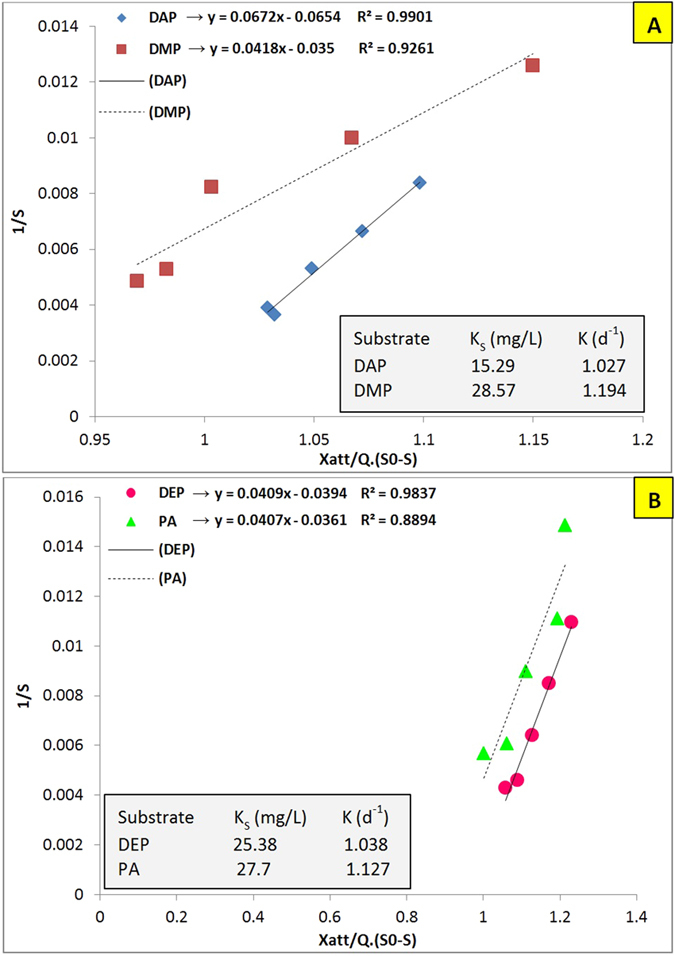
Graphical analysis for the determination of half saturation constant and overall reaction rate for (**A**) DAP and DMP; and (**B**) DEP and PA.

In order to calculate the half saturation constant (K_S_) and overall reaction rate (K), Eq. (7) was plotted in Fig. 5. The obtained overall reaction rate values demonstrate that DMP (1.194 d^−1^) and DAP (1.027 d^−1^) had the maximum and minimum biodegradation rates, respectively. Moreover, the K_S_ values obtained for DMP (28.57 mg/L) and PA (27.7 mg/L) were significantly more than that of DAP (15.29 mg/L); however, these values cannot be directly evaluated as their maximum specific growth rate (μ_m_) and substrate utilization rate (r_su_) are not equal. If the mentioned parameters (μ_m_ and r_su_) for these phthalates were equal, then microbial community for the substrate with lower K_S_ would have more affinity with that substrate in low substrate conditions. In fact, the microbial competition for substrate in the biological process becomes a relevant phenomenon, and the organism with the lowest (K_S_) has the highest affinity with the substrate and will outcompete other organisms present in that culture^54^.

**Figure 6:**
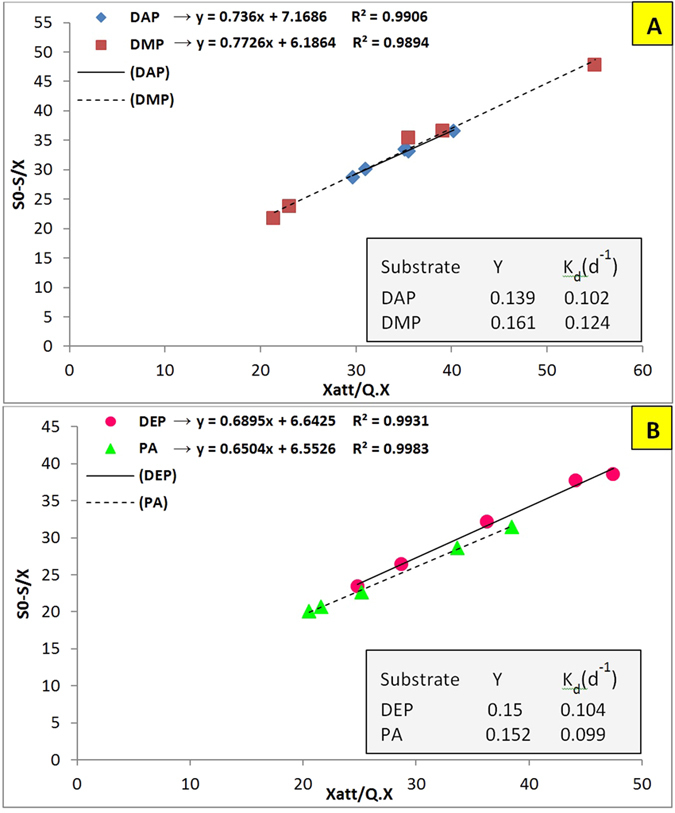
Graphical analysis for the determination of yield coefficient and biomass decay rate for (**A**) DAP and DMP; and (**B**) DEP and PA.

Figure 6 represents yield coefficient (Y) and decay rate (K_d_) of biomass with different substrates. Maximum and minimum yields were observed for DMP and DAP as 0.161 and 0.139, respectively. The yield coefficients with DEP (0.15) and PA (0.152) as substrate were not significantly different. The determined yield coefficients were much lower than aerobic yields reported for conventional wastewater treatments which is typically 0.6^19^. The yield coefficient for aerated submerged fixed-film reactor (ASFFR) with glucose as substrate was reported as 0.63 which is more than four times the yield coefficients calculated in this study. The observed yield coefficients were more than two times other conventional anaerobic processes which are about 0.06^55^. However, other studies reported that yield coefficients for anaerobic conditions can reach 0.3 and higher, which is significantly higher than what was observed in the present study^56^,^57^,^58^. This is an important point of view, because excess sludge treatment and disposal can cause many economic, environmental, and regulation challenges for wastewater treatment plant^59^. Furthermore, the calculated yield coefficients of the present study are close to those of novel technologies such as aerated submerged membrane bioreactor with yield coefficient of about 0.15^60^. According to previous comparisons, AnFFFBR can be introduced as a promising bioreactor, especially because of sludge management. Moreover, decay rates were not considerably different among all phthalates except for DMP. A higher decay rate for DMP may be related to its higher substrate utilization rate which consequently leads to a higher growth rate and, considering the constant available surface area for biofilm growth, more microbial mass may be placed in the inner layer and decay, as it did not access the substrate.

The maximum specific growth rate (μ_m_) estimated for selected phthalates are shown in Table 4. The (μ_m_) values were: 0.193, 0.170, 0.156, and 0.143 d^−1^, which were observed for DMP, PA, DEP, and DAP, respectively. These coefficients confirm the recorded COD results.

## Conclusions

Although all selected phthalates showed acceptable biodegradability, among the four, DMP can be selected as the best substrate due to its highest biodegradability, mineralization, and methane production. Moreover, DMP had the highest values of overall reaction rate and maximum specific growth rate, which confirms that this substrate is more biodegradable than others. According to the results of mathematical modeling, Grau model can be selected as the best model for designing and predicting the AnFFFBRs’ performance due to its strong coefficients of determination. Finally, selected phthalates had similar biodegradation pathways and the main route was the detachment of side alkyl chains (de-esterification).

## Additional Information

**How to cite this article:** Ahmadi, E. *et al*. Performance, kinetic, and biodegradation pathway evaluation of anaerobic fixed film fixed bed reactor in removing phthalic acid esters from wastewater. *Sci. Rep.* **7**, 41020; doi: 10.1038/srep41020 (2017).

**Publisher's note:** Springer Nature remains neutral with regard to jurisdictional claims in published maps and institutional affiliations.

## Figures and Tables

**Table 1:** AnFFFBR’s performance in removing DAP and DMP.

Substrate	DAP					DMP				
Study step	A					B				
Study run	1	2	3	4	5	1	2	3	4	5
Influent phthalate concentration (mg/L)	300	300	300	300	300	300	300	300	300	300
Hydraulic retention time (h)	12	18	24	30	36	12	18	24	30	36
L_org_^1^(gCOD/m^2^.d)	4.275	2.85	2.137	1.71	1.425	3.825	2.55	1.912	1.53	1.275
Attached biofilm mass (TS (mg))	5430	3870	3770	3470	3340	4660	3510	3450	3230	3190
Attached biofilm mass (VS (mg))	3660	2580	2400	2160	1980	3540	2520	2340	2100	1980
VS/TS ratio	0.674	0.666	0.636	0.622	0.592	0.759	0.718	0.678	0.65	0.62
TSS^2^ concentration in effluent (mg/day)	298.8	207.2	174.6	153	129.7	247.8	192	137.3	115.4	86.2
Effluent volatile suspended solids (mg/day)	123.6	83.2	68.4	61	49.2	153.6	117.6	66	53.76	36
SRT^3^ (day)	18.17	18.67	21.59	22.68	25.75	18.8	18.28	25.12	27.98	37.01
Phthalate concentration in effluent TSS (mg/g)	11.9	10.7	8.4	7.7	4.3	6.1	5.2	5.2	4.1	3.4
Effluent phthalate concentration (mg/L)	121.7	104.3	88.4	73.1	49.5	86.2	71.1	61.3	40.3	22.5
Methane production rate (L CH_4_/g COD_rem_) ± SD^4^	0.16 ± 0.016	0.21 ± 0.012	0.27 ± 0.02	0.32 ± 0.016	0.35 ± 0.022	0.24 ± 0.012	0.29 ± 0.01	0.32 ± 0.013	0.37 ± 0.012	0.46 ± 0.009
CH_4_ percentage in biogas (%)	37.6	40.3	51.1	57.3	62.2	42.3	45.3	54	58.9	64.2
Effluent COD concentration (mg/L)	274.4	256.5	188.7	150.3	119.3	205.6	189.4	121.3	100.1	79.5
Phthalate removal% ± SD	59.43 ± 2.23	65.23 ± 2.12	70.53 ± 2.27	75.63 ± 1.7	83.5 ± 1.16	71.26 ± 1.04	76.3 ± 1.89	79.56 ± 1.91	86.56 ± 1.2	92.5 ± 1.44
COD removal % ± SD	51.86 ± 1.97	55 ± 1.71	66.89 ± 1.81	73.63 ± 1.43	79.07 ± 1.25	59.68 ± 1.91	62.86 ± 1.54	76.21 ± 1.52	80.37 ± 1.11	84.41 ± 1.32
TOC removal% ± SD	39.74 ± 1.38	49.53 ± 1.15	58.41 ± 1.45	65.84 ± 1.33	72.46 ± 1.35	46.5 ± 1.66	55.84 ± 1.73	65.44 ± 1.99	74.15 ± 1.58	80.39 ± 1.35

1- Organic loading rate. 2- Total suspended solids. 3-Solids retention time. 4- Standard deviation.

**Table 2:** AnFFFBR’s performance in removing DEP and PA.

Substrate	DEP					PA				
Study step	C					D				
Study run	1	2	3	4	5	1	2	3	4	5
Influent phthalate concentration (mg/L)	300	300	300	300	300	300	300	300	300	300
Hydraulic retention time (h)	12	18	24	30	36	12	18	24	30	36
L_org_ (gCOD/m^2^.d)	4.162	2.775	2.081	1.665	1.387	3.195	2.13	1.597	1.278	1.065
Attached biofilm mass (TS (mg))	5840	4370	4110	3880	3720	4070	3030	3020	2870	2840
Attached biofilm mass (VS (mg))	4080	2940	2700	2460	2280	3000	2220	2100	1920	1740
VS/TS ratio	0.698	0.672	0.657	0.634	0.612	0.737	0.732	0.695	0.669	0.612
TSS concentration in effluent (mg/day)	326.8	227.3	169.3	129.6	112.2	232	162	146	108	93
Effluent volatile suspended solids (mg/day)	164.4	102.4	74.4	55.6	48	148.8	100	83.4	53.3	46
SRT (day)	17.87	19.22	24.27	29.94	33.15	17.54	18.7	20.68	26.57	30.53
Phthalate concentration in effluent TSS (mg/g)	9.7	9.3	8.6	8.1	5.2	5.5	5.3	3.3	3.1	1.8
Effluent phthalate concentration (mg/L)	96.3	82.5	70.6	57.1	36.4	80.7	73.5	62.6	46.8	25.2
Methane production rate (L CH_4_/g COD_rem_) ± SD	0.22 ± 0.01	0.24 ± 0.013	0.28 ± 0.008	0.34 ± 0.012	0.43 ± 0.01	0.19 ± 0.015	0.2 ± 0.013	0.26 ± 0.013	0.37 ± 0.011	0.4 ± 0.01
CH_4_ percentage in biogas (%)	41.1	42.3	52.8	56.1	61.5	43.7	47.7	53.3	58.4	60.3
Effluent COD concentration (mg/L)	233.6	217.2	156.2	117.7	91.3	176.5	163.6	111.3	90.1	67.3
Phthalate removal% ± SD	67.9 ± 1.32	72.5 ± 1.23	76.46 ± 1.13	80.96 ± 1.15	87.86 ± 1.43	73.1 ± 1.87	75.5 ± 1.18	79.13 ± 1.25	84.4 ± 1.4	91.6 ± 1.22
COD removal % ± SD	57.91 ± 1.62	60.86 ± 0.87	71.85 ± 1.04	78.79 ± 1.15	83.55 ± 1.23	58.56 ± 1.59	61.6 ± 1.45	73.87 ± 1.33	78.85 ± 0.83	84.2 ± 1.92
TOC removal% ± SD	45.32 ± 1.62	53.97 ± 1.16	62.58 ± 1.31	70.05 ± 1.41	78.33 ± 1.12	48.12 ± 1.69	56.49 ± 1.34	65.49 ± 1.33	74.79 ± 1.47	81.75 ± 1.29

**Table 3:** Second order (Grau) model coefficient (K_G_).

Study run	1	2	3	4	5
DAP	1.548	2.196	2.361	2.623	2.861
DMP	1.970	2.767	2.980	3.321	3.522
DEP	1.677	2.327	2.534	2.782	3.001
PA	1.804	2.438	2.577	2.819	3.111

**Table 4:** Maximum specific growth rate estimated for AnFFFBR.

Substrate	μ_m_ (d^−1^)
DAP	0.143
DMP	0.193
DEP	0.156
PA	0.170
